# Untuned broadband spiral micro-coils achieve sensitive multi-nuclear NMR TX/RX from microfluidic samples

**DOI:** 10.1038/s41598-021-87247-2

**Published:** 2021-04-08

**Authors:** Hossein Davoodi, Nurdiana Nordin, Hirokazu Munakata, Jan G. Korvink, Neil MacKinnon, Vlad Badilita

**Affiliations:** 1 Institute of Microstructure Technology (IMT), Karlsruhe Institute of Technology (KIT), Hermann-von-Helmholtz-Platz 1, 76344 Eggenstein-Leopoldshafen, Germany; 2 Department of Chemistry, Faculty of Science, University of Malaya, 50603 Kuala Lumpur, Malaysia; 3 Department of Applied Chemistry for Environment, Graduate School of Urban Environmental Sciences, Tokyo Metropolitan University, Tokyo, 192-0397 Japan

**Keywords:** Solution-state NMR, Electrical and electronic engineering, Lab-on-a-chip

## Abstract

The low frequency plateau in the frequency response of an untuned micro-resonator permits broadband radio-frequency reception, albeit at the expense of optimal signal-to-noise ratio for a particular nucleus. In this contribution we determine useful figures of merit for broadband micro-coils, and thereby explore the parametric design space towards acceptable simultaneous excitation and reception of a microfluidic sample over a wide frequency band ranging from ^13^C to ^1^H, i.e., 125–500 MHz in an 11.74 T magnet. The detector achieves 37% of the performance of a comparably sized, tuned and matched resonator, and a linewidth of 17 ppb using standard magnet shims. The use of broadband detectors circumvents numerous difficulties introduced by multi-resonant RF detector circuits, including sample loading effects on matching, channel isolation, and field distortion.

## Introduction

Nuclear magnetic resonance (NMR) is a powerful and versatile analytical tool due to its capability to address and analyse with exquisite chemical specificity each isotope exhibiting a unique magnetic moment. Consequently, NMR methods can be applied to almost all elements in the periodic table. Building upon this feature, NMR is able to offer correlated information either within the chemical shift range of the same isotope (homonuclear) or from different species (heteronuclear), therefore being capable to deliver information related to the structure and dynamics of a molecular system^1,2^ with atomic resolution. This unique characteristic allows the simultaneous detection of multiple nuclei either by parallel^3,4^ or sequential^5^ acquisition, performing decoupling experiments^6,7^, or employing indirect detection methods, such as polarization-transfer techniques^8,9^, to study low gyromagnetic, i.e, less sensitive nuclei.

A multitude of NMR detection hardware variants have been developed in order to meet the needs for multi-nuclear NMR detection, either as multi-resonant or as broadband topologies^10^. Multi-resonant circuits provide tuning and matching at different frequencies utilizing additional capacitors and inductors^11–20^. Despite their popularity, these circuits suffer from low power transfer efficiency compared to their single resonant counterparts. This is attributed to the additional current paths introduced by the extra elements^11–15^, or to the power dissipation in those elements^16–20^. In the receive mode, this issue translates to higher noise level and/or lower signal amplitude, therefore lower signal-to-noise ratio (SNR). Moreover, the resonant frequencies of these circuits are sensitive to the environment and sample loading, meaning that a small perturbation in the environment of the coil can un-match the coil, additional effort being required in order to re-match it. Modifying these circuits in order to capture a different resonant frequency is a cumbersome task, which ends up either in reconstructing the circuit or replacing the circuit elements.

Another approach for multi-nuclear NMR detection is a multi-coil topology, where each coil is tuned and matched at one or multiple frequencies^21–27^. The additional hardware and the cross-talk between the coils are the main issues to be addressed when employing this topology. The challenge encountered when using a multi-coil structure for multi-nuclear detection originates from the overlap of different fields-of-view (FOV) of the participating coils. The efficiency of coherence transfer or decoupling methods is directly influenced by the FOV overlap. At the same time, FOV overlap among different coils is synonymous with a cross-talk among those coils and power leakage. The leaked power reduces the efficiency and limits the effective dynamic range of the low noise amplifier (LNA). Although all coils in a multi-coil structure should share the same FOV, their cross-talk should be minimised. In order to compensate for the isolation issue, extra trap elements are required to decouple the coils. Due to their non-ideal nature, trap circuits introduce noise and power loss, thus degrading the performance of the system as a whole. The orthogonality requirement between the detector coil and the external magnetic fields introduces further challenges to the experimental setup. Besides, the outermost coil, being expanded as a result of additional coils, suffers from low filling factor.

NMR miniaturisation is a well-recognised technique to enhance sensitivity for mass-limited samples by employing miniaturised coils. However, this approach posses a new challenge in multi-resonant circuits and multi-coil configurations. The multi-resonant circuits are bulky compared to the miniaturised coils. Therefore, the interconnections between the circuit elements and the coil exhibit an impedance comparable to the coil impedance. This results in partial power dissipation in these interconnections and additional noise, which degrade both the power delivered to the coil in the excitation mode of operation and the SNR of the receive mode. One solution for the power transfer efficiency constraint is to make the peripheral circuit more compact, i.e., shortening the tracks. The penalties to be paid are the additional static magnetic field distortions introduced by the peripheral circuit, and the coupling between the inductors of those circuits and the micro-coil. The other solution is to use inductive coupling to another coil in order to supply power to the detecting micro-coil. In this case, magnetic field distortions can be eliminated by keeping the supply coil and its circuit away from the FOV of the detecting micro-coil, nevertheless the power transfer efficiency is lower than in the case of wired connection. This is mainly due to the inductive coupling being smaller than unity and the resistance of the supply inductor, resulting in power loss and additional noise. Several studies have tackled the issue of employing micro-coils for multi-nuclear NMR applications, maintaining both spectral resolution and SNR^23,28–30^.

While the above-mentioned topologies rely on resonating detection schemes, several alternative approaches have considered the challenge of multi-nuclear NMR detection in a continuous, wide bandwidth^31–36^. These coils offer a detection bandwidth comparable to the central frequency of the detection band (e.g. measuring ^1^H and ^13^C at 11.7 T, central frequency is 315 MHz and bandwidth is 375 MHz). A widely studied subcategory of wideband detectors are delay and transmission lines with lumped and distributed elements, respectively^31–34^. In this case, the detector is a two-port element, in a form of $\pi$- or T-network of inductors and capacitors, and is terminated to its characteristic impedance at both ports. The inductive elements in the network are exploited as Faraday induction NMR detection elements. A secondary subcategory is the one-port detector^35,36^, i.e., coils being terminated to the ground at one end, as opposed to two-port element delay/transmission line coils. This coil does not require any external tuning matching elements.

In an important paper on the topic of broadband NMR detectors, Fratila et al.^36^ have reported a planar spiral micro-coil that operates in a wide frequency range between 61 and 400 MHz, being able to observe most NMR-active nuclei at 9.4 T with remarkable mass sensitivity. A book chapter by Anders and Velders^37^ clarified the effect of the mismatch between the coil and electronics by studying the signal transfer between them. In spite of the exquisite performance of this detector, the literature still lacks an in-depth analysis of the operational principles of broadband planar spiral coils from an electrical engineering perspective, with NMR application as a key design goal.

This paper bridges the gap in understanding and adopting the broadband coils in NMR applications by providing a thorough fundamental study of working principles accompanied by characterisation results. The discussion also covers the analysis of the parameters to be tuned by the NMR engineer in order to optimise the overall performance of a broadband spiral coil detector and presents the limitations related to the rest of the existing signal chain of the commercial NMR system influencing the broadband performance.

The broadband detector introduced and analysed in this work consists of a miniaturised spiral coil, an RF connection port, and a microfluidic channel. The study starts with introducing the figures of merit for an NMR detector and continues with simulating and optimising the performance of the broadband detector considering these figures of merit at different frequencies. These are compared and discussed with reference to the figures of merit for tuned/matched detectors as an evaluation of the broadband detector. The theoretical discussion and optimisation in the two different regimes (broadband and tuned/matched) are concluded by presenting the optimum coil design in each regime and calculating the penalty to be paid for a broadband detector. However, the general framework of the present study is not limited to the spiral coils and can be applied to any particular coil geometry. We also report on the fabrication procedure for the broadband spiral detector using standard microfabrication techniques. The experimental evaluation of the broadband detector is presented using: (1) NMR experiments at seven different frequencies corresponding to ^1^H (500 MHz), ^19^F (471 MHz), ^31^P (202 MHz), ^7^Li (194 MHz), ^11^B (160 MHz), ^23^Na (132 MHz), and ^13^C (126 MHz) at 11.74 T to verify the broadband performance; (2) ^1^H MRI experiment on a water phantom to evaluate the RF field distribution; (3) impedance measurements (given in “Supplementary Information”). Normalised limit-of-detection (nLOD) is reported for the broadband detector at different frequencies and compared with the state of the art. In addition, the discussion ends with listing the challenges associated with the broadband coils and suggestions are given for further performance enhancement.

## Results

**Figure 1:**
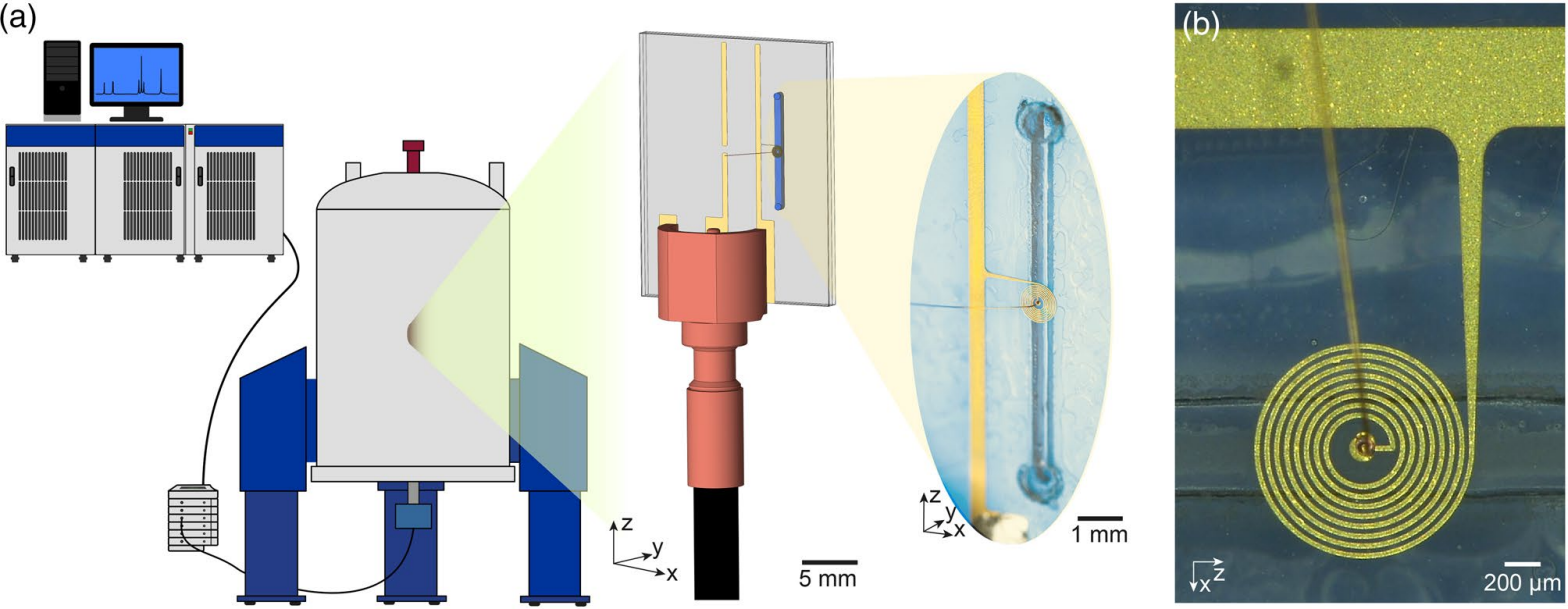
(**a**) NMR experiment setup consisting of a vertical-bore superconductive magnet together with the probe, LNA block, and electronic racks for signal processing. The detector, being mounted on the NMR probe is directly connected to the LNA block using a coaxial cable. The sample is loaded in the microfluidic channel of the detector. (**b**) The broadband coil, wirebond, and the microfludic channel underneath.

Figure 1 shows the NMR setup consisting of a superconducting magnet, pre-amplifier block (also known as low noise amplifier, LNA), and further spectrometer electronics (excitation source, signal processing chain, etc). A probe places the detector and (in this case) the sample inside the magnet. A coaxial cable connects the coil to the LNA block. Although the coil is not matched to ${50}\,\Omega$, both the characteristic impedance of the coaxial cable and the input impedance of the LNA stage are ${50}\,\Omega$, similar to standard NMR systems.

Borrowing from the classical description of NMR, once the sample is placed inside the static magnetic field $B_{0}$, a bulk magnetisation is created along the direction of the magnetic field. By applying a radio-frequency (RF) magnetic field (${\text {B}}_{1}$) for time $\tau$, in a direction perpendicular to the static field, the magnetisation is rotated to an angle of $\theta =\gamma B_1 \tau$, where $\gamma$ is the gyromagnetic ratio of the isotope to be studied. Once the excitation ${\text {B}}_{1}$ is silenced, the magnetisation returns to equilibrium, precessing around $B_0$ axis at the Larmor frequency, $\omega _L=\gamma B_0$. The precessing magnetic moments induce a voltage in the NMR coil. According to the principle of reciprocity^38^, the voltage induced into the coil by an elementary magnetisation ($M_0$) depends on the magnetisation itself and on the RF field generated by the coil at that specific magnetisation location for a unit current applied to the coil ($S(x,y,z,t)={\partial (\frac{B_{1}(x,y,z)}{i} \cdot M_0(x,y,z,t))}/({\partial t})$). The total voltage being induced in the coil is the integral of these elementary voltages over the sample volume ($v_{\text {FID}}(t)=\int _{\text {sample volume}} S(x,y,z,t)\,dv$). The noise spectral density of the micro-coils is usually the thermal noise of the coil expressed by $v_{{\text {n,coil}}}=\sqrt{4 k_B R_{\text {coil}} T}$ where $k_B$ is the Boltzmann constant, $R_{\text {coil}}$ is the coil resistance, and *T* is the coil temperature.

### NMR figures of merit

In order to understand the coil performance under different tuning/matching conditions, two main figures of merit are considered: excitation efficiency and SNR. They describe the coil performance in both excitation and reception modes.

#### Excitation efficiency

The excitation efficiency is defined as the frequency of the nutation signal collected for 1 W excitation power. The excitation source has a predefined output impedance, usually ${50}\,\Omega$. Therefore, optimum power transfer can be achieved by *matching* the coil impedance to the impedance of the excitation source. Deviations from this matching condition decrease the excitation efficiency.

In a conventional probe, the coil is connected to the coaxial cable through a passive tuning and matching network. In its simplest form, this circuit consists of two capacitors. At the resonant frequency, the impedance of the tuning/matching circuit together with the coil, seen from the coaxial cable port, equals the characteristic impedance of the cable (${\text {Z}}_{0}$). Therefore, the maximum power transfer occurs at this specific frequency. Proper matching conditions being fulfilled and at the resonant frequency, half of the power is consumed in the output impedance of the excitation source and half is dissipated in the coil and the tuning/matching circuit, neglecting the dissipation in the cable. As the quality factor of the capacitors is usually high ($> 1000$), almost all power dissipation in the circuit happens in the coil (typically with $Q < 100$). Therefore, the excitation efficiency at the resonant frequency can be calculated as shown in Table 1. In these equations, $B_1/i$ represents the average RF magnetic field at the sample region per unit applied current to the coil.

For a broadband coil, wideband performance is achieved by removing tuning/matching. As the result of mismatch, the amplitude of the current flowing in the coil for a certain applied power depends on the impedance of the coil ($Z_{\text {coil}}$), and hence the excitation efficiency differs from the tuned/matched case as indicated in Table 1.

**Table 1:** NMR figures of merit for two different working regimes, applicable to a conventional tuned/matched coil, and a broadband coil. In all four cases, the figure of merit should be maximised in order to have the best performance.

Figures of merit	Tuned and matched	Broadband
Excitation efficiency (ef)	$\gamma \frac{1}{\sqrt{2 R_{\text {coil}}}} B_{1}/i$	$\gamma \frac{\sqrt{2 Z_0}}{\left| Z_{\text {coil}}+Z_0 \right| } \, B_{1}/i$
SNR	$\frac{v_{\text {FID}}}{\sqrt{F} v_{\text {n,coil}}}$	$\frac{v_{\text {FID}}\frac{Z_0}{\left| Z_0+Z_{\text {coil}} \right| }}{\sqrt{v_{\text {n,coil}}^2\frac{Z_0^2}{\left| Z_0+Z_{\text {coil}}\right| ^2}+v_{n,Z0}^2 \frac{F-1}{4}}}$

#### SNR

SNR is a more complex parameter containing several terms. Some of these terms are predefined at the coil design level, i.e., the properties of the sample and nuclei being investigated, the static magnetic field, and the sample volume or concentration. In the reception mode, an ideal tuning/matching network transfers both the voltage induced into the coil and the noise spectral density of the coil by an amplification factor of $\sqrt{{Z_0}/{R_{\text {coil}}}}$ at the resonant frequency and maintains SNR constant. Neglecting the noise contribution of the coaxial cable and the inter-connections, as well as the power dissipation in these elements, the SNR at the input of the LNA equals the intrinsic SNR of the coil. Another amplification step at the LNA brings the signal to a sufficient level for further processing. During the amplification of the input signal, extra noise is introduced by the LNA. This extra noise is usually defined by the noise factor (*F*) of the LNA. The SNR at the resonant frequency and at the output of the LNA is also given in Table 1. According to the Friis formula for cascaded noise factor^39^, the noise introduced by the LNA is dominant and the additional noise generated by successive processing steps can be ignored. As discussed by Massin et al.^40^, at the coil level design and for a given sample and certain ${\text {B}}_{0}$, the intrinsic SNR can be enhanced by increasing the RF magnetic field amplitude at the sample region per square root of the resistance of the coil through proper selection and design of the coil geometry based on the sample.

For a broadband coil in receive mode, the intrinsic SNR of the coil is the same as in the tuned/matched case. Considering the resonant frequencies of the transmission line far from the working frequencies and neglecting the power dissipation in the transmission lines and the noise contribution of the cable and connections, the SNR at the input of the LNA is also the same as the intrinsic SNR of the coil, as long as the cable is terminated to its characteristic impedance at the LNA side. Assuming the same LNA in both tuned/matched and broadband cases and approximating the input referred noise of the LNA with just a voltage source, the output SNR can be derived according to Table 1, where $v_{\text {n,Z0}}$ represents the noise corresponding to the characteristic impedance ($Z_0$).

An intrinsic characteristic of a coil operating in a broadband regime is the unavoidable degradation of the overall performance of the system compared to tuned/matched operation, due to the mismatch between the coil and the other excitation/detection circuits. In the excitation mode, lack of tuning/matching results in a longer excitation pulse for a given applied power or, equivalently, higher excitation power for a given excitation pulse length. On the other hand, since the $v_{n,Z0}$ is usually higher than the thermal noise of the coil, lack of the tuning/matching affects the overall detection performance. These drawbacks can be compensated by accumulating multiple scans. Therefore, in a broadband coil, a compromise in sensitivity is exchanged for an increased flexibility to operate over multiple frequencies. The challenge of the coil designer is to optimise the performance as a function of application and hence the required bandwidth.

### Optimisation of detector geometry

The spiral coil was defined on the backside of a ${100}\,{\upmu {\text {m}}}$-thick glass substrate and the coil was formed from gold. Gold was a microfabrication constraint, since the available copper wirebonding machine requires gold bonding pads. The design parameters defining the coil are: inner radius ($r_{\text {in}}$), number of turns (*n*), the width of the tracks (*w*), the spacing between the tracks (*s*), and the thickness of the tracks (*h*). The inner turn of the coil was connected to the corresponding port using a wirebonded bridge. The wirebond requires a $100 \, \upmu {\text {m}}$ pad at the centre of the coil, thus the inner radius of the coil was set to ${125}\,{\upmu {\text {m}}}$ as a good compromise between a small sample volume and high RF field. The values for *s* and *w* were optimised based on the analytical formula for the magnetic field generated at the coil axis, discussed in the “Supplementary Information”. For this case, $w+s={40}\,{\upmu {\text {m}}}$ results in the highest $B_1$ field at the sample region. In order to use the cross-section of the tracks efficiently, the thickness and the width of the tracks should be more than twice the skin depth at the working frequencies. The skin depth of gold at 100 MHz is about ${7.5}\,{\upmu {\text {m}}}$, thus *w*, *h*, and *s* were selected to be ${20}\,{\upmu {\text {m}}}$.

Simulations and performance analysis were carried out at three different frequencies. These frequencies were selected based on the Larmor frequency values of the most popular isotopes, listed in a recent publication of our group^10^, i.e, 500 MHz, 200 MHz, and 150 MHz. These simulations were performed assuming the sample volume as a cylinder with ${500}\,{\upmu {\text {m}}}$ diameter and ${500}\,{\upmu {\text {m}}}$ height, positioned co-axially with respect to the coil.

Figure S1a depicts the profile of the RF magnetic field for a unit current ($B_1/i$) applied to the ports of the spiral coil. The results are plotted at 500 MHz; however, the relative values are very similar at the frequencies tested. By increasing the number of turns, $B_1$ field homogeneity within the sample volume (dashed blue rectangle) is enhanced, while the $B_1$ field intensity is a maximum for the coil with 25 turns. Simulated physical parameters of the coil (impedance and average RF magnetic field per unit current) as a function of the number of turns at different frequencies are plotted in Fig. S1b. It is important to note that a coil with 29 turns has a self-resonance at about 500 MHz. For values of *n* smaller than 29, the inductance and the resistance of the coil increase with the number of turns. The average magnetic field at the sample region also increases with the number of turns until *n* reaches the self-resonance value. Then, the average magnetic field drops dramatically because of the inter-turn capacitive links bypassing the inductive links.

The simulation results were further processed in order to extract the nutation signals and to gain a better understanding of the performance of different designs at different frequencies. The nutation signals for different numbers of turns, plotted in Fig. S1c, reveal an optimum number of turns in which the excitation efficiency is maximum. Unlike the nutation frequency, which depends on the excitation efficiency, the decay rate of the nutation signal corresponds to the RF field inhomogeneities and is independent of power transfer between the coil and electronics. These nutation signals also confirm RF field homogeneity enhancement with the number of turns.

**Figure 2:**
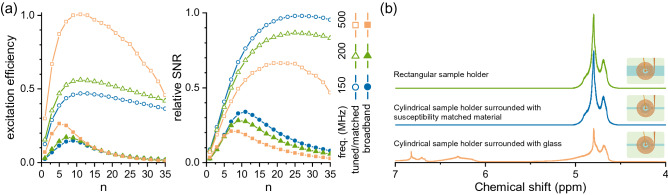
The results of both RF and ${\text {B}}_{0}$ simulations (presented in Fig. S1) were post-processed to calculate the figures of merit and NMR spectra of the spiral coils. The coil geometry is defined by an inner diameter of ${250}\,{\upmu {\text {m}}}$, a track width of ${20}\,{\upmu {\text {m}}}$, a track spacing of ${20}\,{\upmu {\text {m}}}$, and a track thickness of ${20}\,{\upmu {\text {m}}}$. (**a**) The figures of merit are calculated for both tuned/matched and broadband coils, and for a sample of ${500}\,{\upmu {\text {m}}}$ in diameter, ${500}\,{\upmu {\text {m}}}$ in height, a ${100}\,{\upmu {\text {m}}}$ gap between the sample and the coil, and as a function of the number of turns. The plots are normalised to their maximum value. (**b**) NMR spectra of the optimised broadband coil (n=8) for three different sample holders, at 500 MHz corresponding to the ^1^H Larmor frequency at 11.74 T. The distortions in the peak shape correspond to the susceptibility mismatch between the sample, coil, air, and the sample holder. The volume magnetic susceptibility for different materials are assigned according to values reported elsewhere^41,42^. These values are as follows: $\chi _{\text {air}}=0$, $\chi _{\text {glass}}=-11.059\times 10^{-6}$, $\chi _{\text {water}}=-9.035\times 10^{-6}$, $\chi _{\text {gold}}=-34\times 10^{-6}$, $\chi _{\text {copper}}=-9.63\times 10^{-6}$.

The figures of merit were also calculated based on the simulation results and for two different scenarios, i.e, tuned/matched case and broadband performance, according to the formulae presented in Table 1. The results, presented in Fig. 2a, are normalised with respect to the maximum value for a convenient comparison. The results clearly show the existence of particular values for the number of turns for which excitation efficiency and SNR are maximised. Further quantitative comparisons of the optimum geometries are available in Table S1.

To calculate the SNR, the noise figures of the LNAs of ^1^H channel and X-nuclei channel were assumed 1 dB and 2 dB, respectively, to be consistent with the commercially available LNAs introduced in the “Methods” section. The desired values for the number of turns (indicated by $n_{max}$ in Table S1) are different for the two different operation modes (tuned/matched and broadband) and for each operating frequency. These values decrease with frequency. Considering these results, the NMR engineer has to decide on a certain penalty to be paid when defining one single coil for the entire frequency range. A compromise in the optimum number of windings was determined by averaging the calculated optimal number of winding values over both the excitation efficiency and SNR figures of merit, at the evaluated frequencies for each configuration ($3 \ n_{max}$ values for each figure of merit presented in Table S1). This resulted in 17 windings for the tuned/matched case and 8 for broadband case. A detailed evaluation of the optimal coil for each configuration is given in Table S1.

Table S1 also summarises the cost for the broadband performance with respect to the tuned/matched case. It is important to note that, although the excitation efficiency suffers considerably from mismatch, it can be easily compensated by employing a longer pulse ($\sim 3$–$5\times$) or a higher applied power ($\sim 9-20\times$). Accordingly, the SNR of the broadband detector is 32–37% of its tuned/matched counterpart. This implies that the total cost, to be paid for a broadband detection, is $\sim 10\times$ more averaging in comparison to an optimised single resonant detector. Nevertheless, for a multi-nuclear application, the SNR degradation, stemming from tank/trap circuits, should be included in order to have a fair comparison (not considered in this work). One solution to enhance the overall SNR is to use LNAs with better noise performance. For instance, a noise figure of 0.5 dB enhances the SNR up to $\sim$ 50%.

Another important feature of an NMR spectrum is its spectral resolution. Apart from the sample properties, i.e., transverse relaxation time ${\text {T}}_{2}$, spectral resolution depends on the ${\text {B}}_{0}$ distribution at the detection zone and is independent of tuning and/or matching. The NMR detector designed based on the optimised broadband coil geometry was further investigated using susceptibility simulations, aiming for an optimised microfluidic channel geometry to minimise ${\text {B}}_{0}$ perturbations at the coil field of view (FOV). NMR spectra were constructed summing all the signals collected from elementary samples (extracted from ${\text {B}}_{1}$ simulations) and considering the relative static field distribution (extracted from ${\text {B}}_{0}$ simulations). In order to minimise the interference of the connector and metallic structures with ${\text {B}}_{0}$ distribution in the sample region, both the coil and the channel have been shifted towards one edge of the chip. Fig. 2b shows NMR spectra simulated for three different sample holders.

According to ${\text {B}}_{0}$ field patterns, given in Fig. S1d, a sample volume with a circular footprint (${500}\,{\upmu {\text {m}}}$ diameter) supplied with channels of ${100}\,{\upmu {\text {m}}}$ width structured in glass results in severe distortions. Replacing the glass with an arbitrary susceptibility-matched medium enhances the spectral resolution and, therefore, the quality of the spectrum. Residual imperfections are caused by the metallic structures, i.e, the coil and its corresponding tracks. To remove material boundaries near to the detection volume, the channel width was increased to ${500}\,{\upmu {\text {m}}}$ with the cost of increased dead sample volume. In this case, the simulation shows a spectrum which is similar to the susceptibility-matched case. The amplitude of the signal in the rectangular channel is slightly higher since a larger amount of sample is contributing to the signal.

### Experimental analysis

#### NMR spectroscopy

**Figure 3:**
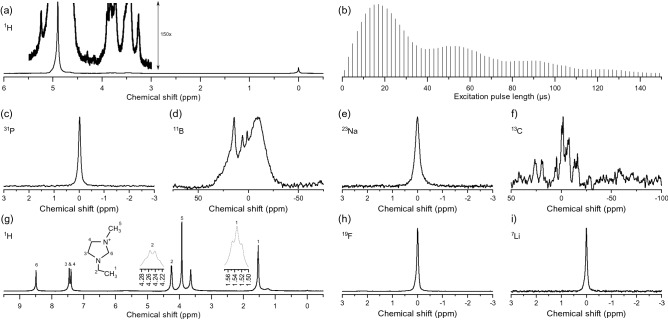
NMR experiment using the optimised NMR detector to observe different nuclei: (**a**) ^1^H NMR spectrum (at 500 MHz) of the aqueous sample. The inset shows the same spectrum with enlarged amplitude. (**b**) ^1^H nutation signal. (**c**) ^31^P NMR spectrum (at 202 MHz) of the aqueous sample. (**d**) ^11^B NMR spectrum (at 160 MHz) of the aqueous sample. (**e**) ^23^Na NMR spectrum (at 132 MHz) of the aqueous sample. (**f**) ^13^C NMR spectrum (at 126 MHz) of the aqueous sample. (**g**) ^1^H NMR spectrum (at 500 MHz) of the electrolyte sample. (**h**) ^19^F NMR spectrum (at 471 MHz) of the electrolyte sample. (**i**) ^7^Li NMR spectrum (at 194 MHz) of the electrolyte sample.

The optimised detector, depicted in Fig. 1 and fabricated according to the procedure given in “Methods” section, was tested to detect seven different nuclei. For this purpose, two different samples (aqueous and battery electrolyte material) have been investigated. The aqueous sample containing ^1^H, ^31^P, ^11^B, ^23^Na, and ^13^C isotopes is a good representative for the biological samples, whereas the battery electrolyte sample demonstrates a different class of NMR applications. Experiments on the battery electrolyte sample were performed at frequencies corresponding to ^1^H, ^19^F, and ^7^Li isotopes, the results being presented in Fig. 3.

NMR results on the aqueous sample clearly demonstrate the advantage of employing a broadband coil, since no adjustment to the detector is made during the experiments. Considering the constraint of multi-resonant micro-coils, e.g., the efficiency of the trap/tank circuits and the geometrical restrictions imposed by the limited space inside the magnet bore, it is only the broadband coil approach that allows the detection of five or more nuclei, without interrupting the measurements for detector modification.

The ^1^H spectral linewidth, measured for the TSP signal (0 ppm), is 8.3 Hz. Although this value is generally accepted for the micro-samples, the main obstacle to further improve the spectral resolution is the sample chamber not being at the isocentre of the magnet and the limited current of the shim channels. The shims of the commercial wide-bore spectrometer are constructed and calibrated for macroscopic samples and coils, therefore the range of shim currents can not compensate for very local field inhomogeneities, especially if the sample volume is not at the isocentre of the magnet (the case here). The ^1^H nutation signal is depicted in Fig. 3b. According to these results, the maximum NMR signal intensity was achieved at an excitation pulse of $\tau =$ ${17}\, \upmu \text {s}$.

^31^P and ^23^Na experiments each demonstrate a single peak corresponding to the phosphate buffer solution (PBS), prepared using the sodium phosphate salts. The ^11^B experiment shows a wide signal which is attributed to the boric anhydride ${\text {B}}_2\text {O}_{3}$ content in the glass substrate (D 263 T eco). In addition, multiple signals were observed as a result of boric acid chemical reaction with glucose. The overlapping signal corresponds to the borate–glucose complex molecule^43,44^. ^13^C experiments could also successfully reveal the glucose content although with a relatively low SNR which is because of its low receptivity and is inevitable in a direct detection scheme.

In the battery electrolyte sample, ^1^H signal originated from the EMI protons in the solvent. The triplet signal at ${\sim 1.50}$–1.56 ppm is assigned to the proton at position 1, followed by quartet signal at ${\sim 4.22}$ ppm to ${\sim 4.48}$ ppm for carbon 2. A singlet, observed at ${\sim 3.80}$ ppm is assigned to the methyl protons at carbon 5. Protons at carbon 3 and 4 are symmetric and have the same doublet signals at the chemical shift in the higher field from ${\sim 7.40}$–7.50 ppm. Finally, a singlet signal was observed at ${\sim 8.50}$ ppm from the proton at carbon 6. ^7^Li spectrum shows the lithium in the LiTFSI and ^19^F content in both LiTFSI and TFSI was detected as a single peak in the ^19^F spectrum. The measurement results are summarised in Table 2.

Figure S2a–e compare the digital twins of the NMR spectra, constructed from the simulation results, with the actual spectra collected from different nuclei. For an easier comparison, the simulation results were adjusted to include all experimental parameters, e.g., number of averages and bandwidth. The results have also been scaled to have the same noise level. Two major differences between the simulation and measurement results can be observed: (1) the broader and distorted simulated spectra report on severe static field distortion as discussed in Fig. S1d. In the measurement results, the shim adjustment was employed to compensate for these distortions and, as a result, the spectral resolution has been improved. (2) The measurement results exhibit lower SNR with respect to the simulation. This is because of the noise contribution and power loss imposed by the elements in the signal flow chain. The SNR resulted from both simulated and measured spectra are listed in Table 2. The difference between the simulation and measurements is more prominent for low gyromagnetic-ratio nuclei because of their lower intrinsic SNR. Figure S2f compares the ^1^H nutation signals calculated from measurements and simulations. The decay rate of the nutation signal matches perfectly with the simulation results. The only difference between these results is the $\pi /2$ pulse lengths ($\tau _{\pi /2}=$ ${17}\,{\upmu \text {s}}$ in the experimental and ${13}\,{\upmu \text {s}}$ in the simulation) which is attributed to the power dissipation in the connectors, the solder points, the coaxial cable, and the excitation source.

**Table 2:** NMR experiment results at seven different frequencies prove the broadband performance of the detector. SNR values were scaled to their single-scan equivalent before calculating nLOD. Here, $nLOD=\frac{3 n_s \sqrt{t_{acq}}}{SNR_1} \left( \frac{B_0}{14.1} \right) ^{7/4}$, where $n_s$ is the number of spins, $t_{acq}$ is the acquisition time, $SNR_1$ is the single-scan SNR, and $B_0$ is the static magnetic field. The excitation pulse width was defined based on the maximum amplitude of the nutation curve. SNR is calculated in the frequency domain (the ratio between the signal and the rms noise). The excitation pulse width is the same for almost all the nuclei, due to the fact that the impedance of the coil changes linearly with frequency (being dominated by the inductance) and hence the current in the coil compensates for the reduced gyromagnetic ratio. The simulated SNR values extracted from the ’digital twins’ of the spectra given in Fig. S2.

Isotopes	Aqueous sample					Electrolyte sample		
	^1^H	^31^P	^11^B	^23^Na	^13^C	^1^H	^19^F	^7^Li
Excitation power (W)	1	1	1	1	1	1	1	1
Excitation pulse width (ms)	17	17	17	15	17	17	17	15
Number of averages	512	2048	2048	32768	32768	256	128	512
Relaxation delay (s)	10	4	0.1	5	0.1	10	10	1
Measured SNR	4295	112.5	–	54	7.7	830.5	597.9	82.5
Simulated SNR	5428	147	–	142	–	–	777	126
nLOD ($\text {nmol}\, \text {s}^{1/2}$)	88	135	–	1025	–	–	36	66

As a unified method for reporting NMR detector sensitivity, we report the frequency-domain normalised limit of detection (nLOD) at 14.1 T magnetic field^45,46^, summarised in Table 2. According to these results, nLOD of the proposed broadband detector is comparable to the other single resonant detectors reported elsewhere (around ${50}\, \text {nmol}\, \text {s}^{1/2}$ for 100 nl sample volume)^46–48^. It should be noted that by overcoming the limit of shim currents, the nLOD values would be remarkably enhanced. For this purpose, one can consider susceptibility matching techniques^49,50^ or shim-on-chip structures^51^, which are perfectly compatible with the current detector.

These results are additionally limited by ${50}\,\Omega$ input impedance of the LNA and characteristic impedance of the cable. By employing an LNA with higher input impedance and design the coil accordingly, the results would be further improved. As a trend in micro-NMR, several studies have reported transceiver circuits being integrated to the coil^52–55^. The synergy between broadband NMR detectors and ASICs (application specific integrated circuits) will play a big role in future micro-NMR.

#### MRI experiments

**Figure 4:**
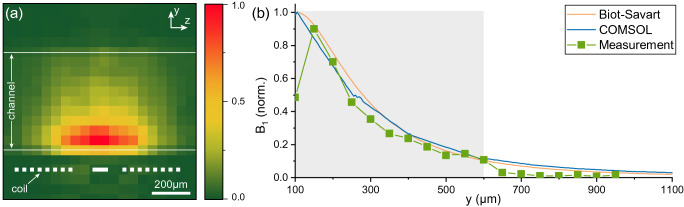
(**a**) Map of ${\text {B}}_{1}$ field (the component of the RF field being normal to ${\text {B}}_{0}$) generated by the coil at the sample region extracted from the MRI experiments. (**b**) Measured ${\text {B}}_{1}$ field, generated by the coil at its axis, is in good agreement with the simulation results and analytical expression derived from Biot–Savart formula (Eq. S1). The MRI results are valid just within the sample volume (indicated with the gray rectangle). The mismatch at $y={0}\,{\upmu {\text {m}}}$ is a measurement error because of partial sample at the boundary voxels, as shown in (**a**).

The RF field pattern, extracted from ^1^H MRI experiments, is given in Fig. 4. This profile, achieved based on the procedure given in the “Methods” section, is compared with the simulation results and analytical expression derived from Biot–Savart formula (discussed in “Supplementary Information”). These results are in good agreement, confirming the accuracy of the simulations, and validate the simplifying assumptions made for the Biot-Savart equation.

## Discussion

In a standard NMR experiment with a coil tuned to a single frequency, removing the coil and sample and tuning the coil to a secondary frequency is time consuming, affects the static field homogeneity, and causes relative dislocation of the sample and the coil. Thus, for experiments requiring multi-nuclear detection, a multi-resonant coil would be a better option. However, the trap/tank circuits introduce power loss and degrade both excitation efficiency and SNR in multi-resonant coils. It is even more challenging when adapting such circuits to micro-coils, since the micro-coils exhibit relatively small impedances and therefore the power loss in the accompanying circuits is considerable. An alternative solution is based on the broadband concept. This report provides a new layer of understanding by an in-depth, systematic study of the broadband coils, considering their different aspects. This contribution introduces a toolbox to accurately analyse and predict the performance of the detectors, either in broadband or in tuned/matched mode, using a ’digital twin’ of the NMR figures of merit. In addition to the theoretical discussions, we have optimised and exploited a broadband spiral coil. According to the simulation results, the optimum design is more than 32% as efficient as a single resonant coil, in terms of SNR. It should be noted that the lower SNR values can be compensated by more averaging. In a fair comparison, the efficiency of the broadband micro-coil should be compared to a multi-resonant micro-coil (in this case seven resonances) considering all previously-mentioned sources of decreased efficiency.

The optimised geometry has been fabricated using standard microfabrication techniques. Microfabrication has also enabled us to tailor the sample chamber and the microfluidic channel for the coil, and to form a hybrid system as the NMR detector. This detector has been experimentally evaluated at seven different frequencies. Besides, ^1^H MRI experiments have been performed to verify the simulations. The normalised limit of detection extracted from the measurement results proves that this detector, despite being broadband, has a performance that is comparable to the micro-detectors reported in an earlier review^46^, bearing in mind that those detectors are single resonant and cannot work at frequencies other than ^1^H. It is worth mentioning that, even for a multi-resonant macro-coil, detection of seven different frequencies without any interruption to modify the circuit seems to be a non-solved problem. One shall also notice that the performance of the broadband coil is not limited to seven nuclei presented here, but the very same coil can be exploited for nuclei within the frequency range tested. Moreover, the performance of this detector can be further enhanced by integrating other features such as shim-on-chip features or integrating ASICs to amplify the collected signal locally and immunise it to the signal loss by the impedance mismatch between the commercial LNAs and the coil.

## Methods

### Finite element method (FEM) simulations

In order to evaluate and optimise the coil geometry, the commercially available COMSOL MultiPhysics 5.5 (COMSOL AB, Sweden) was used. The RF and susceptibility studies have been carried out using RF and AC/DC modules, respectively. The relative tolerances of the RF simulations were set to $1\times 10^{-4}$ and relative tolerances of the susceptibility were $1\times 10^{-10}$. The simulation results were processed in MATLAB R2018a (MathWorks Inc., USA) to generate the ’digital twin’ of NMR and nutation spectra and calculate the NMR detector figures of merit. To calculate the nutation spectra and the figures of merit, ${\text {B}}_{0}$ was assumed to be perfectly homogeneous. The NMR spectra were generated at $\pi /2$ flip-angle and with no additional static field correction. Permeability values were assigned to different materials based on the values discussed elsewhere^41,42^.

### Microfabrication process

**Figure 5:**
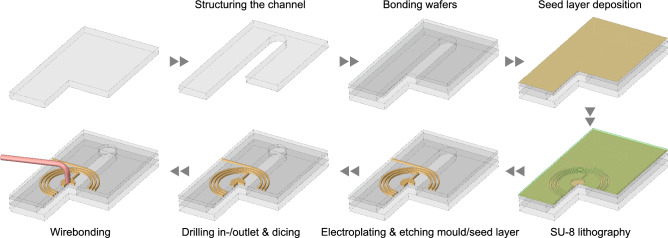
Fabrication process started with structuring the channel in the middle wafer. The process was continued with bonding three wafers together with SU-8 (not shown here) and coating the top side of the assembly with the seed layer. Using UV-lithography, a mould was patterned on the top side and the metallic structures were deposited. After removing the mold and the seed layer, the inlet and outlets were drilled with laser and the assembly was diced into individual chips. The process was concluded by connecting the centre pad of the coil to its corresponding track.

As depicted in Fig. 5, the broadband detector consists of an assembly of three D 263 T eco wafers bonded together. The middle wafer is ${500}\,{\upmu {\text {m}}}$ thick. The microfluidic channels were structured in this wafer with a nanosecond laser (PIRANHA ACSYS). The wafers were bonded employing ${5}\,{\upmu {\text {m}}}$-thick not-crosslinked, spincoated, SU-8 photoresist layers as the glue and employing a compression-bonding machine (EVG510 EV Group) with 5 kN force and at ${55}\,^{\circ }\text {C}$ for ${4}\text { h}$. The assembly was exposed with UV-light (${300}\text { mJ cm}^{-2}$ of ${365}\text { nm}$) followed by post-exposure bake (${95}\,^{\circ }\text {C}$ for 1 h) to enhance the chemical and mechanical stability of the bond. The ${100}\,{\upmu {\text {m}}}$ thick uppermost wafer was coated with chromium/gold seed layers (20/60 nm). The coil and access pads and tracks were patterned using standard SU-8 lithography process to produce the mould and gold-electrodeposition (${20}\,{\upmu {\text {m}}}$). The ${500}\,{\upmu {\text {m}}}$ thick lowermost layer seals the microfluidic channel and maintains the mechanical stability of the structure. The inlet and outlet were lasered through the top wafer and the assembly was diced into individual chips. The centre pad of the coil was wirebonded to its corresponding track using an automated ball-wedge Cu wirebonder (3100 ESEC, Switzerland).

### Probe head manufacturing

An MMBX connector was soldered to the detector to connect the chip to the coaxial cable (HUBER + SUHNER, Switzerland) bypassing all the tuning/matching circuits embedded in the conventional probe. The cable was 2 m in length and had an MMBX plug at one end and an N-type connector at the other end. It had an attenuation of less than 0.605 dB per meter and could tolerate continuous power of more than 54 W. The detector was mounted on a PMMA adaptor which fixes the detector on the probe.

### Sample selection and preparation

#### Aqueous sample

D-Glucose ($\ge$ 99.5%), 13-C labelled D-Glucose ($\ge$ 99.5%), 3-(trimethylsilyl)- propionic-2,2,3,3-${\text {d}}_{4}$ acid sodium salt (99.9 atom %, TSP), deuterium oxide ${\text {D}}_{2}\text {O}$ (99.9 atom % D), phosphate buffer saline (PBS) tablets, boric acid (ACS reagent, $\ge$ 99.5%), and sodium dihydrogen phosphate (BioReagent, for molecular biology, anhydrous, $\ge$ 98%) were purchased from Sigma-Aldrich without further purification.

The acqueous test sample was prepared as 500 mM 13-C labelled D-Glucose, 500 mM boric acid, 500 mM ${\text {NaH}}_{2}{\text {PO}}_{4}$ and 250 mM TSP dissolved in 3 mL of a 500 mM PBS solution (pH 5.5, using PBS tablets) prepared using 50% ${\text {H}}_2{\text {O}}$ and 50% ${\text {D}}_2{\text {O}}$.

#### Battery electrolyte sample

1-Ethyl-3-methylimidazolium bis(trifluoromethanesulfonyl)imide (EMI-TFSI) is one of the major ionic liquids having attracted attention as novel solvent due to its high thermal stability, non-volatility and extremely low vapor pressure. These characteristics offer advantages as green solvents (recycling ability and easy product recovery) over traditional organic solvents. Furthermore, its viscosity, hydrophobicity, dielectric constant, and other physicochemical properties are tunable by changing the combination of anionic and cationic constituents. The use of ionic liquids is spreading into various fields of applications, such as organic synthesis and extraction. In electrochemical energy conversion, ionic liquids are in the spotlight as non-flammable electrolytes to provide safer lithium-ion batteries, in which Li salt is added into the ionic liquid as Li^+^-ion conductor. Lithium bis(trifluoromethane-sulfonyl)imide (LiTFSI) is a typical example for the EMI-TFSI class^56–58^. For NMR experiments, Lithium bis(trifluoromethane-sulfonyl)imide (LiTFSI) salt was dissolved in 1-ethyl-3-methylimidazolium bis(trifluoromethanesulfonyl)imide (EMI-TFSI) to make 1 M solution. Both chemicals were purchased from Tokyo Kasei Co., Ltd (Tokyo, Japan) and used without further purification.

### NMR/MRI experiments

The NMR/MRI were carried out using a 11.74 T Avance III Bruker NMR system (Bruker BioSpin, Rheinstetten, Germany) equipped with a Micro5 micro-imaging NMR probe. The sample was loaded in the microfluidic channel and the gradient sleeve was inserted. The probe together with the detector and gradient were inserted inside the magnet so that the middle axis of the microfluidic channel was along the *z*-axis of the magnet, whereas the excitation RF field is along the nominal *y*-axis of the magnet frame of reference (see Fig. 1).

For the ^1^H measurement, the signal was routed through a narrow-band pre-amplifier (^1^H LNA MODULE 500, Bruker BioSpin, Rheinstetten, Germany) with 1 dB noise figure. X-nuclei signals were directed through a broadband pre-amplifier (XBB19F 2HS MODULE 500, Bruker BioSpin, Rheinstetten, Germany) with 2 dB noise figure. NMR experiments were performed using TopSpin 3.5pl2, the operating and processing software for Bruker NMR spectrometers. The applied power was 1 W and shimming was performed manually on the ^1^H spectrum and was kept the same for all the other experiments. The temperature was maintained at ${30}\;^{\circ }\text {C}$ using the water cooling system embedded in the gradient. All NMR spectra were measured using an excitation pulse width reported in Table 2.

#### General procedure

In all cases, the resulting signals were cumulated and multiplied with an exponential function equivalent to a specified amount of line broadening, followed by Fourier transformation. Unless otherwise specified, base line and phase adjustments were performed automatically.

#### ^1^H NMR

The spectra acquired from the aqueous sample and electrolyte sample consisted of 512 scans and 256 scans, respectively. 20,480 data points were collected for each scan with 20 ppm spectral width for the aqueous sample and 15 ppm spectral width for the battery electrolyte sample. The relaxation delay was 10 s in both experiments. 0.3 Hz line broadening applied. ${\text {B}}_{1}$ field homogeneity was evaluated using the nutation experiment on the aqueous sample employing 76 single scans at 1 W applied power with an increment of 2 ms in the pulse length. The relaxation delay between two consecutive scans was set to 15 s. The data points were integrated and graphed as a bar plot to reduce the distortions introduced by relaxation damping or ${\text {B}}_{0}$ inhomogeneities.

#### ^19^F NMR

The spectrum consisted of 128 scans. 20,480 data points were collected for each scan with 20 ppm spectral width, while the relaxation delay was 10 s. 10 Hz line broadening applied.

#### ^31^P NMR

The spectrum consisted of 2048 scans. 30,720 data points were collected for each scan with 60 ppm spectral width while the relaxation delay was 4 s. 5 Hz line broadening applied.

#### ^7^Li NMR

The spectrum consisted of 512 scans. 5120 data points were collected for each scan with 20 ppm spectral width while the relaxation delay was 1 s. 2 Hz line broadening applied.

#### ^11^B NMR

The spectrum consisted of 32,768 scans. 10,240 data points were collected for each scan with 250 ppm spectral width while the relaxation delay was 0.1 s. 100 Hz applied. Phase adjustment was done manually.

#### ^23^Na NMR

The spectrum consisted of 2048 scans. 2048 data points were collected for each scan with 30 ppm spectral width while the relaxation delay was 5 s. 1 Hz line broadening applied.

#### ^13^C NMR

The spectrum consisted of 32,768 scans. 20,480 data points were collected for each scan with 200 ppm spectral width while the relaxation delay was 0.1 s. 10 Hz line broadening applied. Base line adjustment was done manually.

#### ^1^H MRI

MRI experiments were performed using ParaVision 6.0.1 and the aqueous sample. For this purpose, *FLASH* sequences with the following settings were performed: TR/TE—1000/4 ms, slice thickness—${100}\,{\upmu {\text {m}}}$ along the nominal *y*-axis, in-plane resolution—$32\times 32$, Tscan—21 min, FOV—$1.6\times 1.6 \,\text { mm}^{2}$, bandwidth—5.4 kHz, and 64 averages. The excitation pulse length was set to 1.1 ms and the power level was swept from 0.1 to 624.1 mW non-linearly to have a linear sweep over flip angle. In order to determine the exact ${\text {B}}_{1}$ field of each voxel, the collected data were post-processed in MATLAB R2018a (MathWorks Inc., USA) to calculate the nutation spectrum of each voxel. ${\text {B}}_{1}$ map was reconstructed using those nutation spectra.

## Data availability

The datasets generated during the current study are available from the corresponding authors on reasonable request.

## Supplementary information

Supplementary Information.
