# Supplementary material for: Untuned broadband spiral micro-coils achieve sensitive multi-nuclear NMR TX/RX from microfluidic samples
Source: Sci Rep. 2021 Apr 8;11:7798. doi: 10.1038/s41598-021-87247-2 (PMC8032710; doi:10.1038/s41598-021-87247-2)
Supplement: Supplementary file 1 — Supplementary Information. [file 41598_2021_87247_MOESM1_ESM.pdf]

Untuned broadband spiral micro-coils achieve sensitive multi-nuclear NMR TX/RX from microfluidic samples

H. Davoodi, N. Nordin, H. Munakata, J. G. Korvink, N. MacKinnon, V. Badilita

Supplementary information

Coil geometry optimisation

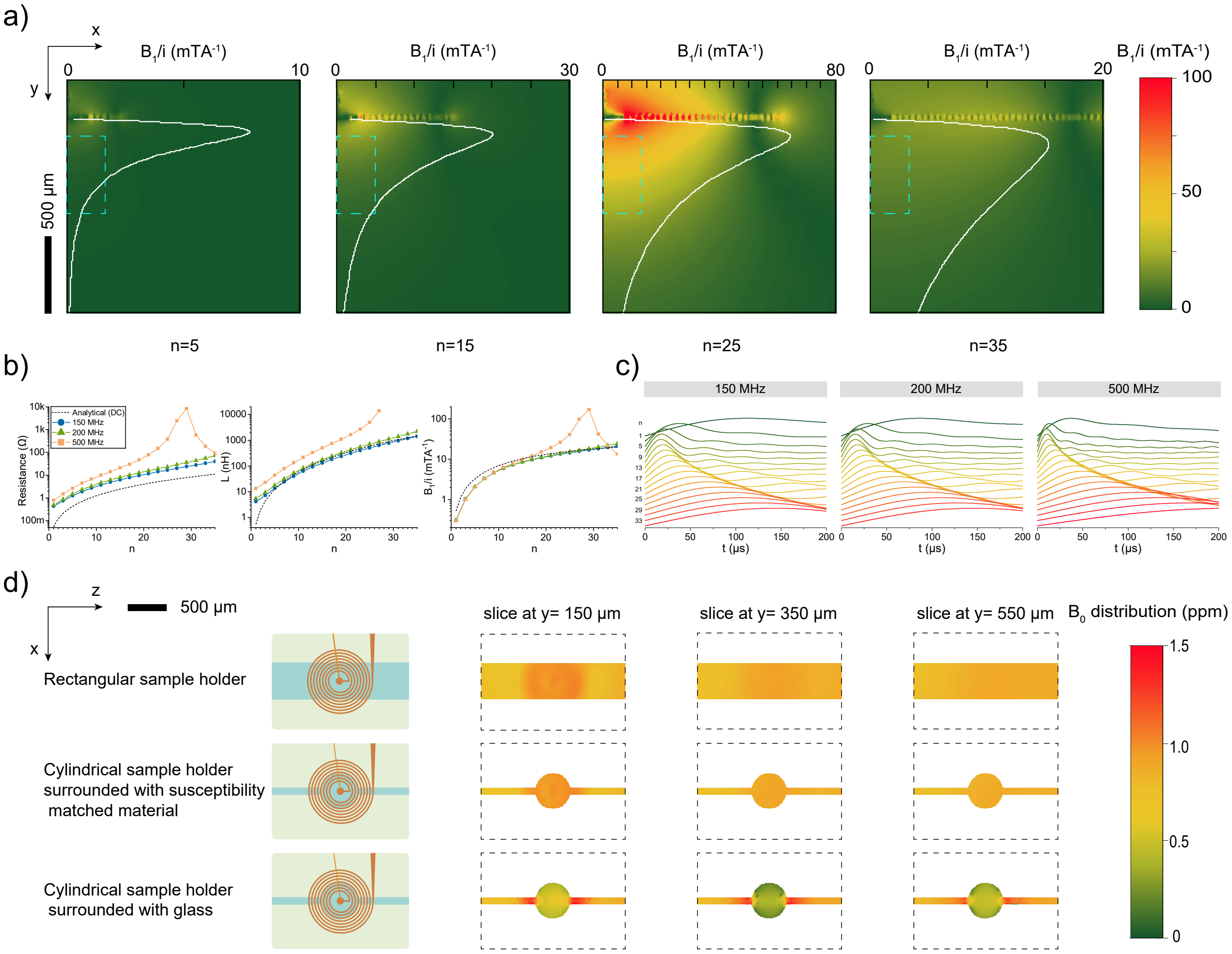

Figure S1. Simulation results using COMSOL for spiral coils (inner diameter=250 µm, track width=20 µm, spacing=20 µm, track thickness=20 µm, sample diameter=500 µm, sample height=500 µm, gap between the sample and the coil=100 µm) with diffrent numbers of turns (n). a) B_1/i at 500 MHz (corresponding to a ^{1}H Larmor frequency at 11.74 T). The dashed rectangle represents the cylindrical sample volume (revolving the figure around the coil axis). The line plot (white curve) together with top x-axis shows the field at the axis of the coil. b) Resistance, inductance, and average B_1/i at three different frequencies and for different numbers of turns. c) Nutation curves of the broadband coils for the predefined sample volume at three different frequencies, for different number of turns, and for a broadband coil. d) B_0 distribution of the optimised coil (n=8) for three different sample geometries and at three different distances from the coil. The susceptibility values were obtained from elsewhere[1,2].

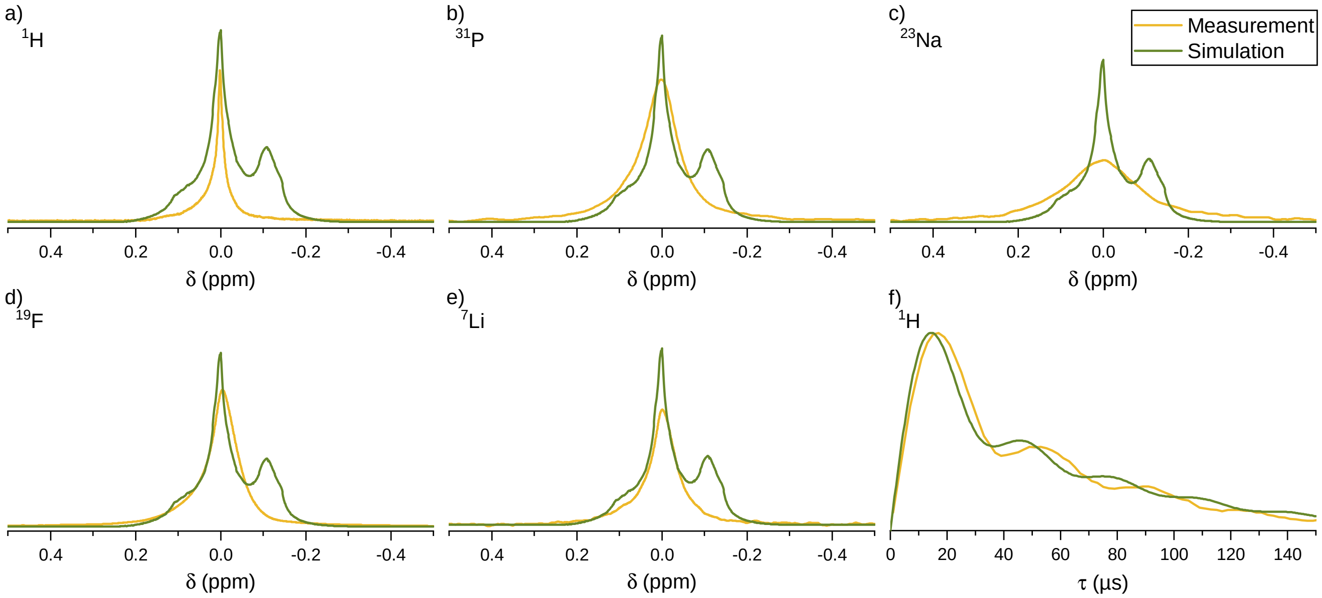

Figure S2. Comparison of the experimental NMR spectra with their 'digital twins'. For the noise calculations, the detection bandwidths were set to their experimental parameters and the temperature was set to room temperature. The spectra were calculated considering a $\pi/2$ excitation pulse. The noise contribution of the sample was ignored and the effect of averaging was considered when generating the digital twins. All the signal paths except the coil and the LNA were assumed to be noiseless. The spectra corresponding to each nucleus are scaled to have the same noise level. The spectral resolution of the experimental spectra was improved using shim adjustments. Therefore, the two distinct peaks of the digital twins merged to one peak in the measurements. The differences in the height of the peaks are attributed to the additional noise and power loss introduced by the sample, interconnections, coaxial cable, and LNA, as well as the noise introduced during signal processing steps. a-c) are based on the data collected from the aqueous sample and d,e) are based on the data collected from electrolyte sample. The results are summarised in Table 2 under measured/simulated SNR. f) compares the nutation signals for ^{1}H signal collected from both measurements and simulations . The mismatch corresponds to the power loss in the excitation path.

Configuration	frequency (MHz)	relative excitation efficiency			relative SNR			n_{opt}
		n_{max}	ef_{max}	ef_{opt}	n_{max}	SNR_{max}	SNR_{opt}	
tuned/matched	150	12	0.47	0.45	25	1	0.94	17
	200	11	0.56	0.54	25	0.89	0.84	
	500	11	1	0.94	19	0.69	0.67	
broadband	150	9	0.15	0.14	11	0.35	0.31	8
	200	7	0.17	0.17	9	0.29	0.27	
	500	5	0.26	0.23	7	0.22	0.21	

Table S1. The number of turns corresponding to the maximum figures of merits in both tuned/matched and broadband configurations and at different frequencies were extracted from the simulation results. The optimum number of turns is calculated by averaging 6 values of n_{max} corresponding to each configuration, respectively. The figures of merit for optimised geometries highlight the total cost to be paid when selecting an optimum number of turns relative to the highest value of the figures of merit. For example, at 500 MHz the optimised tuned/matched coil (n=17) is 2 % less efficient compared to a tuned/matched coil with n=19. The cost for broadband performance can be understood by directly comparing the corresponding values in each configuration. For example, at 200 MHz an optimised broadband coil (n=8) is 0.32× as efficient as the optimised tuned/matched coil (n=17).

Coil characteristics

For a spiral coil working at frequencies far below its self-resonant frequency, the RF magnetic field at the axis of the coil generated for a unit current can be calculated[3] using Biot-Savart law as

$$B_{1_u}(z_o) = \frac{\mu}{2(w+s)}\left[\frac{r}{\sqrt{z_o^2+r^2}} - \frac{r+(w+s)n}{\sqrt{z_o^2+[r+(w+s)n]^2}} + ln\left(\frac{r+(w+s)n+\sqrt{z_o^2+[r+(w+s)n]^2}}{r+\sqrt{z_o^2+r^2}}\right)\right] \tag{S1}$$

where z_o is the distance between the observation point and the centre of the coil, and has been assumed much larger than the thickness of the tracks. μ is the magnetic permeability of the sample, r is the inner radius of the coil, and n is number of turns. w and s are the width of the tracks and the separation between them, respectively and are assumed to be constant.

Benchtop measurements

In order to verify the simulations and analyse the coil performance, the impedance of the optimised coil was measured and compared to the simulation results. For this purpose, a test structure containing a coil with the same number of turns as the optimised coil was designed and fabricated together with the detectors as shown in Figure S3. The impedance was measured using a probe station (MPS150, FormFactor Thiendorf, Germany) connected to a Network Analyzer (E5071C, Keysight Stuttgart, Germany) employing a Z-probe (Z0-20-K3N-GS-50, FormFactor Thiendorf, Germany) and WinCal XE software (FormFactor Thiendorf, Germany) as shown in Figure S3. In order to eliminate the parasitic effects of the metallic chunk on the field distribution of the coils and their impedance, it was replaced with a PMMA chunk after calibration. A similar geometry was simulated using COMSOL. The results are presented in Figure S3. These results confirm the accuracy of the simulations for sub-GHz frequencies.

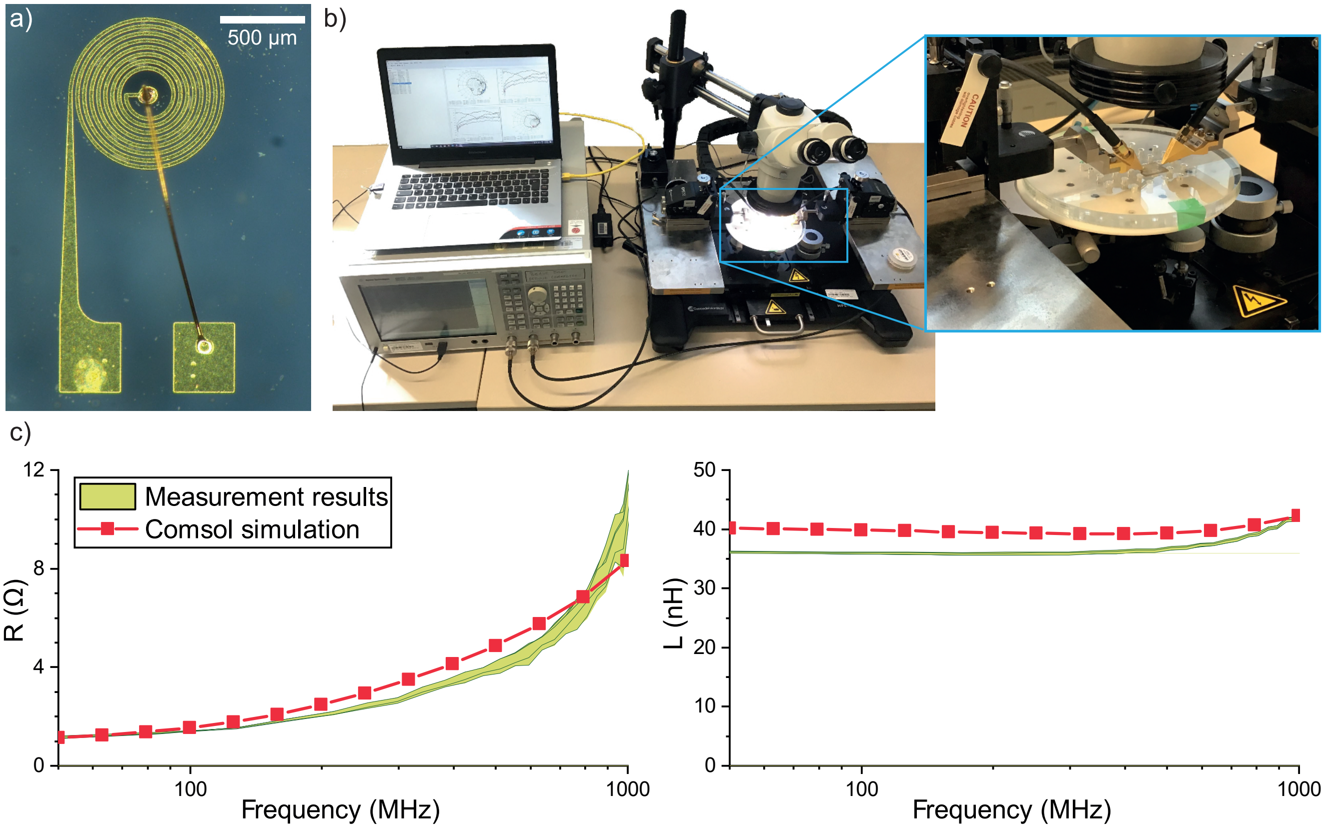

Figure S3. a) Test structure for benchtop RF measurement. b) The impedance measurement setup. c) Impedance of the test structure in comparison with the simulation results.

References

1. Schenck, J. F. The role of magnetic susceptibility in magnetic resonance imaging: MRI magnetic compatibility of the first and second kinds. *Med. Phys.* **23**, 815–850, DOI: 10.1118/1.597854 (1996).

2. Wapler, M. C. *et al.* Magnetic properties of materials for MR engineering, micro-MR and beyond. *J. Magn. Reson.* **242**, 233 – 242, DOI: 10.1016/j.jmr.2014.02.005 (2014).

3. Eroglu, S., Gimi, B., Roman, B., Friedman, G. & Magin, R. L. NMR spiral surface microcoils: Design, fabrication, and imaging. *Concepts Magn. Reson.* **17B**, 1–10, DOI: 10.1002/cmr.b.10068 (2003).
